# Alleviating D-Galactose-Induced Aging in Mice by Modulating Gut-Liver Axis Using *Lactiplantibacillus plantarum* TY-Y10

**DOI:** 10.3390/foods13223618

**Published:** 2024-11-13

**Authors:** Shaoqi Shi, Xiaoxia Li, Feng Zhang, Zhengqiang Jiang, Jing Wang, Liang Zhao, Juan Chen, Xi Shu, Bing Fang, Ping Liu, Jingjing He, Shaoyang Ge, Fuqing Wang, Jie Guo, Yixuan Li, Jie Luo, Ran Wang

**Affiliations:** 1 Key Laboratory of Functional Dairy, Department of Nutrition and Health, Co-Constructed by Ministry of Education and Beijing Government, China Agricultural University, Beijing 100190, China; ssqshishaoqi@163.com (S.S.);; 2 Chongqing Key Laboratory for Industry and Informatization of Probiotic Fermentation Technology in Dairy Products, Chongqing Tianyou Dairy Co., Ltd., Chongqing 401120, China; 3 Key Laboratory of Food Bioengineering (China National Light Industry), College of Food Science and Nutritional Engineering, China Agricultural University, Beijing 100083, China; 4 Research Center for Probiotics, China Agricultural University, Beijing 100083, China; 5 Food Laboratory of Zhongyuan, Luohe 461103, China; 6 Hebei Engineering Research Center of Animal Product, Sanhe 065200, China; 7 Tibet Tianhong Science and Technology Co., Ltd., Lhasa 850000, China; 8 College of Food Science and Technology, Hunan Agricultural University, Changsha 410114, China

**Keywords:** probiotic, aging, Nrf2 signaling pathway, gut microbiota, short-chain fatty acids

## Abstract

Oxidative stress is closely linked to aging. Probiotics, whether viable or heat-inactivated, have shown antioxidant properties; however, their effect and mechanism of action in reducing oxidative stress during aging remains underexplored. This study examined the effects of viable and heat-inactivated *Lactiplantibacillus plantarum* TY-Y10 (*L. plantarum* TY-Y10) on D-galactose (D-gal)-induced aging in mice, aiming to uncover potential anti-aging mechanisms. Mice were induced to age with D-gal injections, then treated with sodium ascorbate (positive control) or varying doses of *L. plantarum* TY-Y10 for eight weeks. After treatment, oxidative stress markers, gut microbiota, and liver health were analyzed. Results showed that *L. plantarum* TY-Y10 decreased malondialdehyde (MDA) and inflammatory markers while increasing antioxidant levels (glutathione, superoxide dismutase, catalase and glutathione peroxidase). Liver damage was reduced, and expression of Nrf2 and related antioxidant enzymes improved. Additionally, *L. plantarum* TY-Y10 enhanced the abundance of short-chain fatty acid-producing bacteria, boosting fecal short-chain fatty acid levels. In short, both viable and heat-inactivated *L. plantarum* TY-Y10 mitigated oxidative stress in aging mice by modulating gut microbiota and activating liver antioxidant pathways through the gut-liver axis.

## 1. Introduction

Aging is a biological process marked by the progressive deterioration of physical and physiological functions, ultimately resulting in mortality [1]. Harman first proposed oxidative damage is a key factor in the aging process in 1956 [2]. Since then, it has been closely associated with the biological mechanisms of aging [3]. Generally, oxidative stress refers to the imbalance between the production of reactive oxygen species (ROS) and the body’s ability to neutralize these reactive molecules with its antioxidant defenses [4]. Excessive ROS oxidizes vital biological macromolecules, including lipids, proteins, and nucleic acids, leading to cellular dysfunction and metabolic abnormality in organs [5]. During the aging process, increasing levels of oxidative stress promote the development of neurodegeneration, cardiovascular disease, cancer, and other chronic disorders [6]. Consequently, the supplementation of antioxidants has been considered an effective strategy for delaying aging and reducing the risk of age-related diseases [7].

The gut microbiota comprises a complex community of microorganisms residing within the gastrointestinal tract, playing a pivotal role in health maintenance and disease modulation through its interactions with host immune and metabolic systems [8]. Research indicates that the composition and functionality of the gut microbiota undergo significant alterations in response to oxidative stress associated with aging. Specifically, there is a decrease in beneficial bacterial genera, such as *Bifidobacterium* and *Lactobacillus*, alongside a rise in pro-inflammatory commensal species, including *Enterobacteriaceae* and *Clostridia* [9]. The disturbed gut microbiota may affect antioxidant activity and contribute to the onset of age-related conditions, including Alzheimer’s and Parkinson’s diseases [10,11,12]. Furthermore, metabolites produced by gut microbes are increasingly recognized for their role in regulating antioxidant and anti-aging signaling pathways in many studies [13,14,15]. Mechanisms related to gut microbiota that influence aging have been recognized as novel targets for antioxidant development.

Probiotics are live microorganisms that are widely used in the formulation of dietary supplements, baked goods, and other functional foods, especially dairy products, the most common probiotic-containing foods [16,17,18]. Probiotics have become a popular intervention in alleviating oxidative stress during aging owing to their ability to modulate gut microbiota by providing live microorganisms [19]. Previous studies have found that *Lactiplantibacillus plantarum* JS19-assisted fermented goat milk can improve the health of the spleen and kidneys while mitigating the hepatocyte oxidative damage in aging mice. Furthermore, *Lactiplantibacillus plantarum* X7022 can relieve memory impairment in aging mice by modulating oxidative stress [20,21]. Studies have also shown that *Lacticaseibacillus paracasei* PS117 exhibits anti-aging effects on cognition and the gut in naturally aging mice [22]. However, the administration of live microorganisms may not always be suitable because some subjects may experience gastrointestinal discomfort and even systemic infections due to probiotics intolerance [23]. Furthermore, probiotics must be kept alive to achieve their intended therapeutic effect if administration is desired. This requirement poses challenges in terms of their storage, industrial processing, maintenance, and application. As a result, more attention has been directed toward exploring the potential therapeutic effects of inactivated bacteria as plausible alternatives to overcome the challenges associated with probiotics [24]. Studies have reported the benefits of heat-inactivated *Lactobacillus* strains, such as enhanced immunity and improved allergies [25,26]. However, studies on probiotics to attenuate oxidative stress and delay the aging process have focused on viable bacteria, and comparatively few studies examining the anti-aging effects of heat-inactivated bacteria. In addition, the efficacy of heat-inactivated *Lactiplantibacillus plantarum* in modulating the gut microbiota needs to be further evaluated to facilitate the study of the mechanism of its effects on delaying aging.

*Lactiplantibacillus plantarum* TY-Y10 (*L. plantarum* TY-Y10, CGMCC No. 24756) has been identified as a potential probiotic with notable health benefits. Our previous in vitro study confirmed it as a strain with high antioxidant activity in both viable and heat-inactivated states. This study aimed to assess the role of viable and heat-inactivated *L. plantarum* TY-Y10 in relieving oxidative stress in vivo and investigate the potential antioxidant mechanism of the *L. plantarum* TY-Y10.

## 2. Materials and Methods

### 2.1. Cultivation of Strains and Preparation of Samples

The *L. plantarum* TY-Y10 strain utilized in this study was initially isolated from traditionally fermented foods and was preserved in a cryoprotectant solution consisting of 12% (*w*/*v*) skim milk and 10% glycerol to maintain its viability. To prepare the strain for further experimentation, it was first inoculated into De Man, Rogosa and Sharpe (MRS) broth at a 2% (*v*/*v*) inoculum ratio and incubated at 37 °C. The strain underwent two sub-culturing cycles to ensure robust growth. Following incubation, the bacterial cultures were subjected to centrifugation at 4500× *g* for 10 min to pellet the cells. The resulting bacterial pellets were then washed three times with a saline buffer to remove residual media and contaminants. The washed pellets were subsequently freeze-dried with trehalose as a cryoprotectant to produce viable bacterial powder. To generate heat-inactivated bacterial powder, the freeze-dried cells were subjected to thermal treatment at 80 °C for 20 min for use in further experiments. Both the viable and heat-inactivated bacterial powders were then resuspended in normal saline to prepare them for intragastric administration in the study.

### 2.2. Experimental Design and Grouping

In this study, we purchased and used six-week-old male BALB/c mice, weighing between 17 and 22 g, and designated as Specific pathogen Free (SPF) grade (catalog number T002726). All procedures involving these animals adhered to the guidelines set by the China Agriculture University Experimental Animal Ethics Committee, under protocol number AW42102202-5-1. The mice were acclimated in a controlled environment within a standard and pathogen-free animal facility. The housing conditions were maintained at a temperature of 22 ± 2 °C, with a relative humidity of 50 ± 10%, and a 12-h light/dark cycle. The animals had unrestricted access to standard rodent chow and fresh water throughout the study.

**Figure 1:**
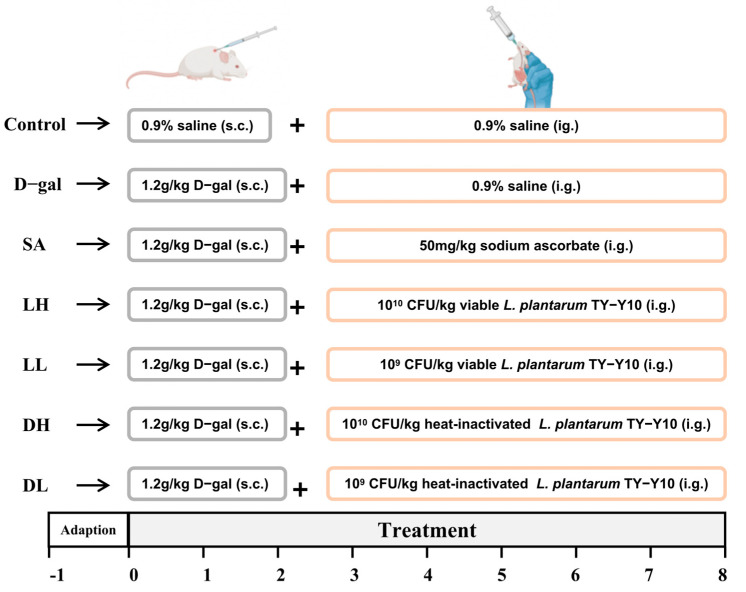
The experimental design of the aging model’s establishment and *L. plantarum* TY-Y10 intervention. The experiment lasted for 8 weeks. From week 0 to 8, except for the control group, mice were injected subcutaneously with D-gal at a dose of 1.2 g/kg every day, and the control group was injected subcutaneously with an equal volume of 0.9% saline. At the same time, mice were gavage-administered 50 mg/kg SA and 10^9^ and 10^8^ CFU/mL of viable and heat-inactivated *L. plantarum* TY-Y10 once a day, and the control group was gavage-administered the same volume of saline. Control, control group; D-gal, model group; SA, positive drug group; LH, high dose of viable and *L. plantarum* TY-Y10; LL, low dose of viable *L. plantarum* TY-Y10; DH, high dose of heat-inactivated *L. plantarum* TY-Y10; DL, low dose of heat-inactivated *L. plantarum* TY-Y10.

After a 7-day acclimation period, a total of 42 mice were randomly allocated to seven experimental groups (n = 6) and groups housed in separate cages (3 mice per cage): a normal control group (control), a group treated with D-galactose (D-gal); a positive control group receiving sodium ascorbate (SA); and four experimental groups receiving either low (LL) or high (LH) doses of viable *L. plantarum* TY-Y10, or low (DL) or high (DH) doses of heat-inactivated viable *L. plantarum* TY-Y10. Experimental procedures are depicted in Figure 1 (created with BioRender.com). The D-gal group was administered daily injections of 0.1 mL of D-galactose at 1.2 g/kg body weight each morning, while the control group received an equal volume of 0.9% normal saline. Both the control and D-gal groups also received 0.2 mL of normal saline daily via oral gavage. The SA group was given 0.2 mL of sodium ascorbate at a concentration of 50 mg/kg. The experimental groups were treated with either viable or heat-inactivated *L. plantarum* TY-Y10 at concentrations of 10^9^ and 10^8^ CFU/mL, administered in 0.2 mL doses. The intervention lasted for eight weeks, a duration consistent with other studies for assessing the antioxidative effects of probiotics in vivo.

During the study, mice were monitored weekly for changes in body weight and food consumption. After the final gavage, the mice were situated in a sterile cage lined with sterile filter paper, and fecal samples were obtained with sterile tweezers immediately after defecation and stored at −80 °C for analysis. Following a 12 h fasting period, all mice were anesthetized with isoflurane, blood was collected via eye socket bleeding, and the mice were euthanized by cervical dislocation. Blood samples were allowed to clot for 2 h and then centrifuged, with the resulting serum stored at −80 °C. Organs such as the heart, liver, spleen, lungs, and kidneys were excised, weighed, and quickly frozen in liquid nitrogen before being stored at −80 °C. The organ coefficient was calculated by dividing the organ weight (mg) by the body weight (g).

### 2.3. Biochemical Analysis

The antioxidant indicators in the serum and liver were measured with reagent kits provided by Nanjing Jiancheng Bioengineering Institute (Nanjing, China). Following the manufacturer’s protocols, we quantified several key biomarkers of oxidative stress and antioxidant defense. Specifically, we measured the concentration of malondialdehyde (MDA), a principal marker of oxidative damage, and glutathione (GSH), a major intracellular antioxidant. Additionally, the activities of crucial antioxidant enzymes were evaluated, including superoxide dismutase (SOD), catalase (CAT), and glutathione peroxidase (GSH-PX). The level of MDA, GSH, SOD, CAT, and GSH-PX was determined using the Thiobarbituric acid (TBA) method, microplate method, hydroxylamine method, visible light method, and colorimetric method. For the analysis of inflammatory responses, serum levels of key cytokines were determined using enzyme-linked immunosorbent assay (ELISA) kits provided by Jiangsu Meimian industrial Co., Ltd. (Yancheng, China). The inflammatory markers assessed included interleukin-6 (IL-6), tumor necrosis factor-alpha (TNF-α), and interferon-gamma (IFN-γ). Each measurement was performed in accordance with the manufacturer’s instructions to ensure accuracy and reliability of the data.

### 2.4. Histological Analysis

Liver tissues were initially preserved in a 4% paraformaldehyde solution to maintain cellular and tissue integrity. Following fixation, the tissues underwent a dehydration process using a series of ethanol concentrations to gradually remove water. Subsequently, the tissues were embedded in paraffin wax to provide structural support for sectioning. Once embedded, the paraffin blocks were sectioned into 5 μm thick slices using a microtome. These tissue sections were then subjected to deparaffinization in xylene, followed by rehydration through a graded series of ethanol solutions to restore the tissue’s original hydration state. The rehydrated sections were stained with hematoxylin and eosin (H&E). Stained sections were imaged with a digital slide scanner (Pannoramic 250, 3DHISTECH, Budapest, Hungary), allowing for high-resolution visualization of the tissue architecture. The captured images were subsequently analyzed to identify and assess any histopathological changes or abnormalities present in the liver tissues.

### 2.5. Quantification of Gene Expression by Real-Time PCR

Total RNA was extracted using the TRIzol/chloroform method. Nanodrop quantification was performed to determine RNA concentration and quality (OD260/OD280), followed by agarose gel electrophoresis and analysis on an Agilent 2100 to check RNA integrity. For cDNA synthesis, total RNA was reverse-transcribed using the 5X All-In-One RT MasterMix. Primer sequences (Table 1) for target genes were designed with Primer Premier software 5.0 and their specificity confirmed using NCBI tools. The primers were then synthesized by Tianyi Huiyuan Biological Technology Co., Ltd. (Beijing, China). Quantitative real-time PCR (RT-PCR) was conducted using the PowerUp™ SYBR™ Green Master Mix kit (Thermo Fisher Scientific Inc., Waltham, MA, USA).

### 2.6. Western Blot Analysis

Liver tissues were initially homogenized using a radioimmunoprecipitation assay (RIPA) buffer, which was supplemented with 1% protease and phosphatase inhibitors to prevent protein degradation. Post-homogenization, the lysates underwent centrifugation at 12,000× *g* and 4 °C for 15 min to separate the soluble proteins from cellular debris. The concentration of total protein in the resulting supernatant was quantified with the Thermo Scientific-Pierce bicinchoninic acid (BCA) protein assay kit (Thermo Fisher Scientific Inc., Waltham, MA, USA). Following protein quantification, samples were subjected to sodium dodecyl sulfate-polyacrylamide gel electrophoresis (SDS-PAGE) for separation based on molecular weight, and then transferred onto polyvinylidene fluoride (PVDF) membranes. The membranes were incubated in a blocking solution of 5% nonfat milk for 2 h at ambient temperature to prevent non-specific binding. Primary antibodies targeting the proteins of interest were applied and incubated overnight at 4 °C. Detection of the proteins was achieved using a highly sensitive chemiluminescent ECL reagent, and the intensity of the protein bands was measured and analyzed using ImageJ software (Version 2.0.0) to assess protein expression levels.

### 2.7. Gut Microbiota Analysis

Fecal samples from mice were collected and immediately stored at −80 °C to preserve microbial DNA integrity. Microbial DNA was extracted from these frozen fecal samples. The extraction process adhered to the manufacturer’s protocol to ensure high-quality and contaminant-free DNA. The integrity and concentration of the extracted genomic DNA were initially evaluated using 1% agarose gel electrophoresis. Further, DNA quality was assessed quantitatively using a NanoDrop spectrophotometer, which provided absorbance ratios (OD_260_/OD_280_) to gauge protein contamination and overall purity. To profile the bacterial community, the V3–V4 hypervariable regions of the 16S rRNA gene were amplified. The amplification was performed using an ABI GeneAmp 9700 PCR thermocycler (Applied Biosystems, Carlsbad, CA, USA), following a standard protocol optimized for bacterial 16S rRNA gene amplification. Post-amplification, the PCR products were purified and quantified. Subsequent sequencing of the amplicons was performed. Following sequencing, the raw sequence data were processed to merge paired end reads. To ensure high data quality, the sequences were subjected to rigorous quality filtering with fastp (version 0.20.0). The resulting high-quality sequences were then clustered into operational taxonomic units (OTUs) using UPARSE software (version 7.1), applying a 97% similarity threshold. This clustering step groups sequences into taxonomic units that represent distinct microbial species or genera. Chimeric sequences, which can distort results due to their artificial nature, were identified and excluded using UCHIME algorithm. Finally, taxonomic classification of each OTU representative sequence was performed using the Ribosomal Database Project (RDP) Classifier (version 2.2). This classifier matches the sequences against the Silva v138 16S rRNA database, providing taxonomic assignments at various levels (e.g., phylum, genus) with a confidence threshold set at 0.7.

### 2.8. Quantification and Analysis of Short-Chain Fatty Acids (SCFAs) in Fecal Samples

SCFAs in fecal samples were quantified using gas chromatography (Agilent Technologies, Santa Clara, CA, USA) following the previously reported procedure [27]. Initially, the fecal samples were treated with 50% sulfuric acid and ether, followed by homogenization and centrifugation to extract the SCFAs. The supernatant obtained was then filtered through a membrane. For analysis, the filtered supernatant was examined using a gas chromatograph (Agilent GC-8860).

### 2.9. Statistical Analysis

The results of this study are presented as the mean ± standard deviation (SD). With significance set at *p* < 0.05, statistical analysis was performed using SPSS software (version 22.0). Group differences were assessed using one-way analysis of variance (ANOVA), followed by Duncan’s multiple range test or Student’s *t* test. Figures were generated by GraphPad Prism 8.

## 3. Results

### 3.1. L. plantarum TY-Y10 Improves the Organ Index in Aging Mice

**Figure 2:**
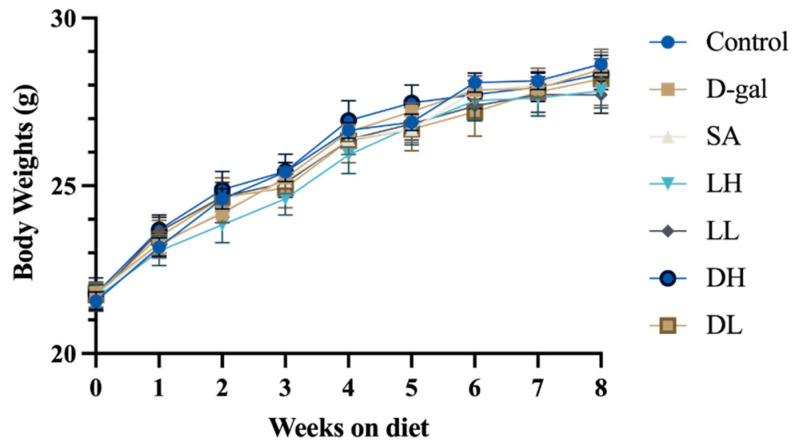
Body weights of mice. The results are presented as means ± SD. Control, control group; D-gal, model group; SA, positive drug group; LH, high dose of viable and *L. plantarum* TY-Y10; LL, low dose of viable *L. plantarum* TY-Y10; DH, high dose of heat-inactivated *L. plantarum* TY-Y10; DL, low dose of heat-inactivated *L. plantarum* TY-Y10. (n = 6).

Initially, we investigated the impact of *L. plantarum* TY-Y10 on body weight in aging mice. As illustrated in Figure 2, the study involved seven groups of 6-week-old mice: control, D-gal, sodium cscorbate (SA), low-dose viable *L. plantarum* TY-Y10 (LL), high-dose viable *L. plantarum* TY-Y10 (LH), low-dose heat-inactivated *L. plantarum* TY-Y10 (DL), and high-dose heat-inactivated *L. plantarum* TY-Y10 (DH). Over the 8-week intervention period, all groups exhibited an increase in average body weight. However, the aging mice treated with D-gal had a marginally lower average weight compared to the control group, though this difference was not statistically significant. In addition, there was no significant difference in the food intake of the mice among the groups.

The organ index serves as a crucial metric for evaluating visceral function from a macroscopic perspective and provides indirect insights into the growth, development, and nutritional status of the animals [28]. As detailed in Table 2, after 8 weeks, the liver organ index in D-gal-induced aging mice was significantly reduced compared to the control group. Treatment with both viable and heat-inactivated *L. plantarum* TY-Y10 notably improved this index in a dose-dependent manner. Additionally, *L. plantarum* TY-Y10 also demonstrated a trend towards improvement in the heart, spleen, and kidney organ indices, although these changes did not reach statistical significance.

### 3.2. L. plantarum TY-Y10 Alleviates Oxidative Stress of Aging Mice

**Figure 3:**
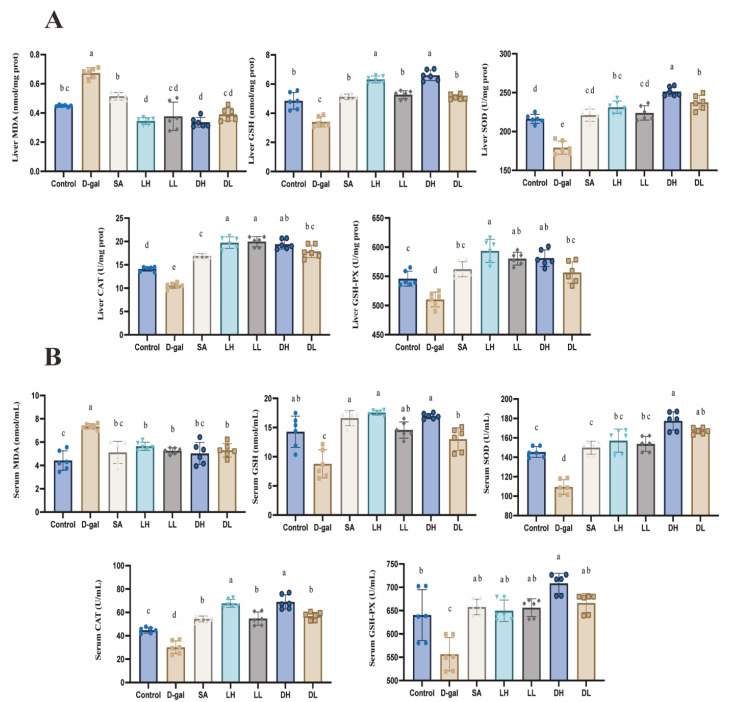
Impact of *L. plantarum* TY-Y10 on oxidative stress levels in serum of aging mice. (**A**) Liver oxidative and antioxidative indexes levels in aging mice; (**B**) serum oxidative and antioxidative indexes levels in aging mice. MDA, malondialdehyde; GSH, glutathione; SOD, superoxide dismutase; CAT, catalase; GSH-PX, glutathione peroxidase. The data are expressed as mean ± SD. Statistical significance was assessed using ANOVA, and differences were considered significant at *p* < 0.05. Groups that differed significantly are indicated by different letters. Control, control group; D-gal, model group; SA, positive drug group; LH, high dose of viable *L. plantarum* TY-Y10; LL, low dose of viable *L. plantarum* TY-Y10; DH, high dose of heat-inactivated *L. plantarum* TY-Y10; DL, low dose of heat-inactivated *L. plantarum* TY-Y10. (n = 6).

The impact of *L. plantarum* TY-Y10 on oxidative stress markers and antioxidant systems in serum and liver tissue of aging mice is illustrated in Figure 3. A comparison between the control and D-gal groups revealed that subcutaneous injection of D-gal significantly elevated MDA levels while reducing GSH content, along with the activities of SOD, CAT, and GSH-PX in both serum and liver. These marked alterations indicate the presence of oxidative stress-induced damage in the aging mice.

Upon treatment with *L. plantarum* TY-Y10, aging mice exhibiting oxidative stress showed a significant reduction in MDA levels in both serum and liver tissues. Concurrently, there was a notable increase in the antioxidant GSH content and the activities of key antioxidative enzymes. The data suggest that *L. plantarum* TY-Y10 effectively mitigates oxidative damage in a dose-dependent manner. Interestingly, the intervention with heat-inactivated *L. plantarum* TY-Y10 tended to show superior improvement in overall antioxidant capacity compared to the viable form of the strain.

### 3.3. L. plantarum TY-Y10 Reduces Serum Inflammation Levels and Protects Liver Tissue in Aging Mice

**Figure 4:**
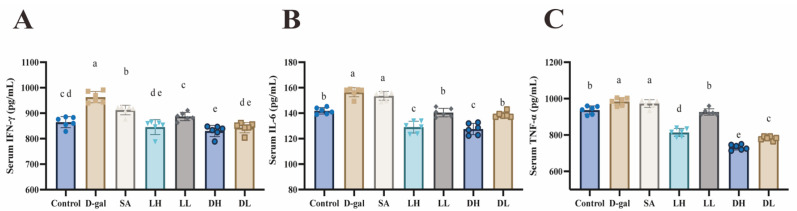
Impact of *L. plantarum* TY-Y10 on inflammation factors in serum of aging mice. (**A**) IFN-γ levels in aging mice serum; (**B**) IL-6 levels in aging mice serum; (**C**) TNF-α levels in aging mice serum. IFN-γ, interferon-gamma; IL-6, interleukin 6; TNF-α, tumor necrosis factor-alpha. The data are expressed as mean ± SD. Statistical significance was assessed using ANOVA, and differences were considered significant at *p* < 0.05. Groups that differed significantly are indicated by different letters. Control, control group; D-gal, model group; SA, positive drug group; LH, high dose of viable *L. plantarum* TY-Y10; LL, low dose of viable *L. plantarum* TY-Y10; DH, high dose of heat-inactivated *L. plantarum* TY-Y10; DL, low dose of heat-inactivated *L. plantarum* TY-Y10. (n = 6).

To determine the impact of *L. plantarum* TY-Y10 on inflammatory responses in aging mice, we analyzed the serum levels of key pro-inflammatory cytokines: IL-6, TNF-α, and IFN-γ, as depicted in Figure 4. The comparison between the control group and the D-gal group revealed that an 8-week administration of D-gal led to a significant elevation in these inflammatory markers, indicating a heightened inflammatory state in the mice. Importantly, treatment with *L. plantarum* TY-Y10, particularly in its heat-inactivated form, substantially mitigated this inflammation. The reduction in serum levels of three pro-inflammatory cytokines observed in the treated groups was statistically significant (*p* < 0.05), suggesting that *L. plantarum* TY-Y10 effectively alleviates inflammation associated with aging.

**Figure 5:**
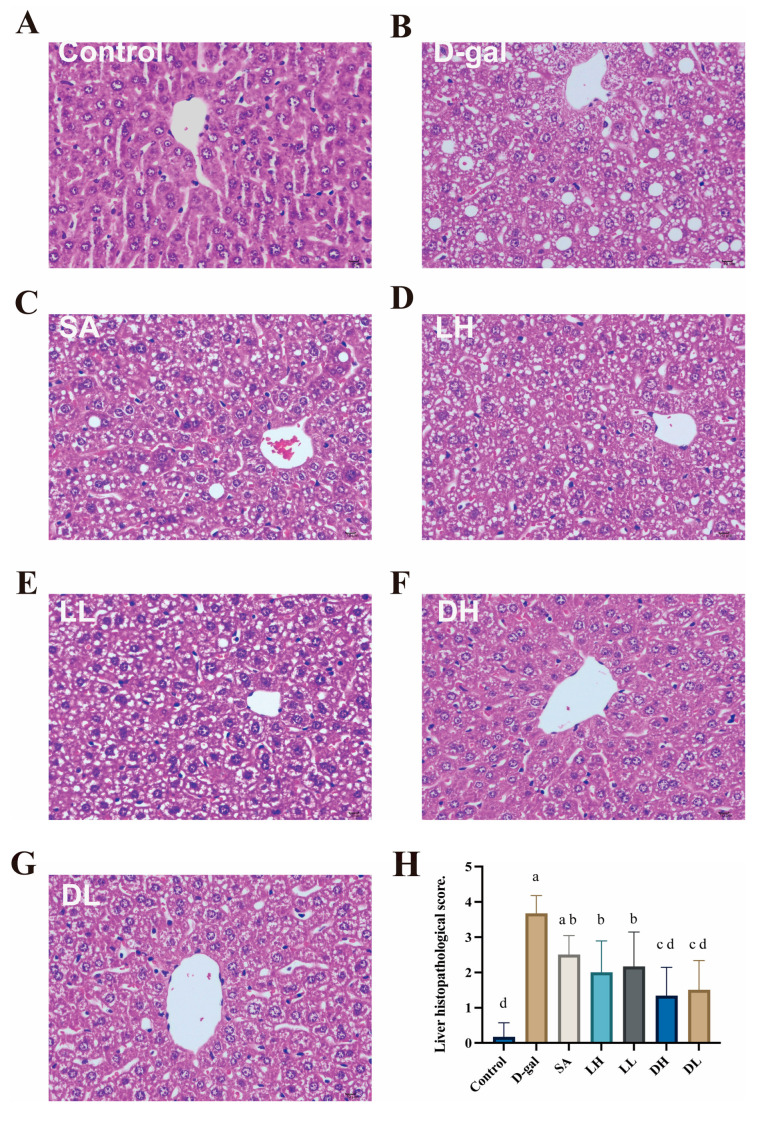
Impact of *L. plantarum* TY-Y10 on liver histopathology of aging mice. (**A**) H&E-stained section of Control group; (**B**) H&E-stained section of D-gal group; (**C**) H&E-stained section of SA group; (**D**) H&E-stained section of LH group; (**E**) H&E-stained section of LL group; (**F**) H&E-stained section of DH group; (**G**) H&E-stained section of DL group; (**H**) pathological scores of liver tissues in different groups. (Original magnification 400×, scale bars are shown with black lines). Statistical significance was assessed using ANOVA, and differences were considered significant at *p* < 0.05. Groups that differed significantly are indicated by different letters. Control, control group; D-gal, model group; SA, positive drug group; LH, high dose of viable and *L. plantarum* TY-Y10; LL, low dose of viable *L. plantarum* TY-Y10; DH, high dose of heat-inactivated *L. plantarum* TY-Y10; DL, low dose of heat-inactivated *L. plantarum* TY-Y10.

Figure 5A–G illustrates the histopathological alterations observed in liver tissues across various experimental groups, as assessed by H&E staining. In the control group (Figure 5A), liver tissues were intact, with hepatocytes arranged radially and regularly around the central vein, showing no signs of cell degeneration, necrosis, or infiltration of inflammatory factors. In contrast, the liver tissues from the D-gal group exhibited pronounced steatosis and vacuolization, indicative of hepatocellular dysfunction and damage [29]. Notably, liver damage observed in the groups treated with *L. plantarum* TY-Y10 showed significant improvement compared to the D-gal group. Specifically, the DH group, which received heat-inactivated *L. plantarum* TY-Y10, displayed a marked reduction in hepatocyte degeneration. These findings suggest that *L. plantarum* TY-Y10 has a protective effect against liver damage and is effective in alleviating inflammation and injury in aging mice.

### 3.4. L. plantarum TY-Y10 Activates Nrf2 Signaling in Aging Mice

**Figure 6:**
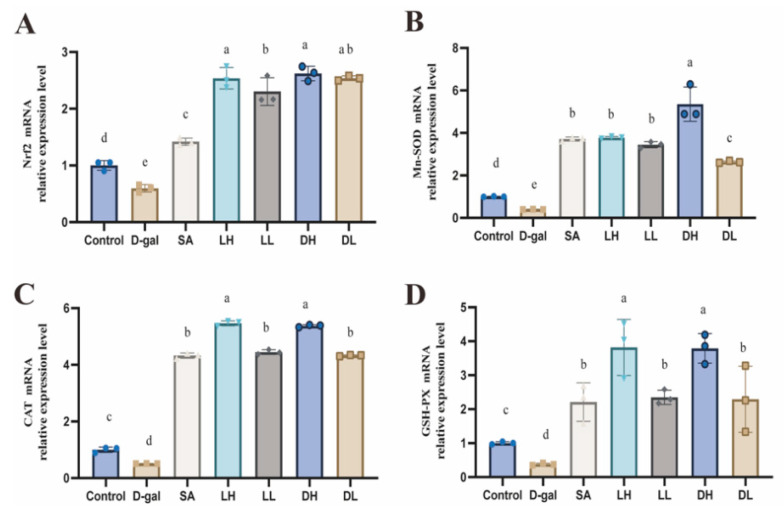
Impact of *L. plantarum* TY-Y10 on mRNA levels of the Nrf2 signaling pathway in aging mice. (**A**) Nrf2 mRNA relative expression level; (**B**) Mn-SOD mRNA relative expression level; (**C**) CAT mRNA relative expression level; (**D**) GSH-PX mRNA relative expression level. Nrf2, nuclear factor erythroid-2. The data are expressed as mean ± SD. Statistical significance was assessed using ANOVA, and differences were considered significant at *p* < 0.05. Groups that differed significantly are indicated by different letters. Control, control group; D-gal, model group; SA, positive drug group; LH, high dose of viable and *L. plantarum* TY-Y10; LL, low dose of viable *L. plantarum* TY-Y10; DH, high dose of heat-inactivated *L. plantarum* TY-Y10; DL, low dose of heat-inactivated *L. plantarum* TY-Y10.

**Figure 7:**
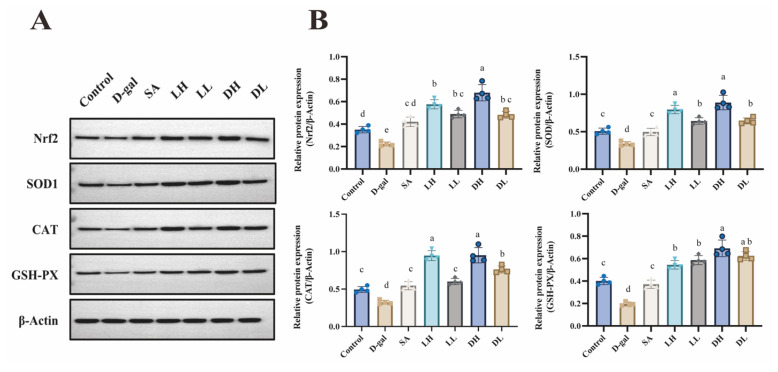
Impact of *L. plantarum* TY-Y10 on protein levels of the Nrf2 signaling pathway in aging mice. (**A**) Nrf2 and its downstream protein detected by Western blot; (**B**) β-Actin as an internal control; Nrf2 and its downstream protein expression were quantified by Image J software. The data are expressed as mean ± SD. Statistical significance was assessed using ANOVA, and differences were considered significant at *p* < 0.05. Groups that differed significantly are indicated by different letters. Control, control group; D-gal, model group; SA, positive drug group; LH, high dose of viable and *L. plantarum* TY-Y10; LL, low dose of viable *L. plantarum* TY-Y10; DH, high dose of heat-inactivated *L. plantarum* TY-Y10; DL, low dose of heat-inactivated *L. plantarum* TY-Y10.

To explore the involvement of the Nrf2 signaling pathway in the protective effects of *L. plantarum* TY-Y10 against oxidative stress in aging mice, we analyzed both mRNA and protein expression levels of key components of this pathway in the liver tissue across different experimental groups. The relative mRNA expression levels of antioxidant enzyme genes, including SOD, CAT, GSH-PX, and nuclear factor erythroid-2 (Nrf2), are illustrated in Figure 6. Additionally, Figure 7 displays the corresponding protein expression levels, providing further insight into the activation of the Nrf2 pathway in response to *L. plantarum* TY-Y10 intervention.

As shown in Figure 6A and Figure 7A, compared with the control group, the relative expression levels of Nrf2 mRNA and protein in the D-gal group were reduced 0.59-fold and 0.63-fold, respectively. This suggests that D-gal induction suppresses the expression of Nrf2 in mice liver. After intragastric administration of *L. plantarum* TY-Y10, the previously downregulated Nrf2 expression (both mRNA and protein) was significantly elevated.

To further verify the effect of *L. plantarum* TY-Y10 on activating the Nrf2-dependent antioxidant signaling pathway, we assessed both mRNA and protein expressions of downstream effectors of Nrf2 in the livers of aging mice, including SOD1, SOD2, CAT, and GSH-PX. As expected, D-gal administration also significantly decreased the mRNA and protein expressions of these downstream effectors, while *L. plantarum* TY-Y10 intervention reversed the above significant changes.

These findings elucidate the mechanism through which *L. plantarum* TY-Y10 ameliorates D-gal-induced oxidative stress injury. Specifically, *L. plantarum* TY-Y10 exerts its protective effects by enhancing the expression of antioxidant enzymes at both the mRNA and protein levels, through the activation of the Nrf2 signaling pathway.

### 3.5. L. plantarum TY-Y10 Reshapes the Gut Microbiota in Aging Mice

**Figure 8:**
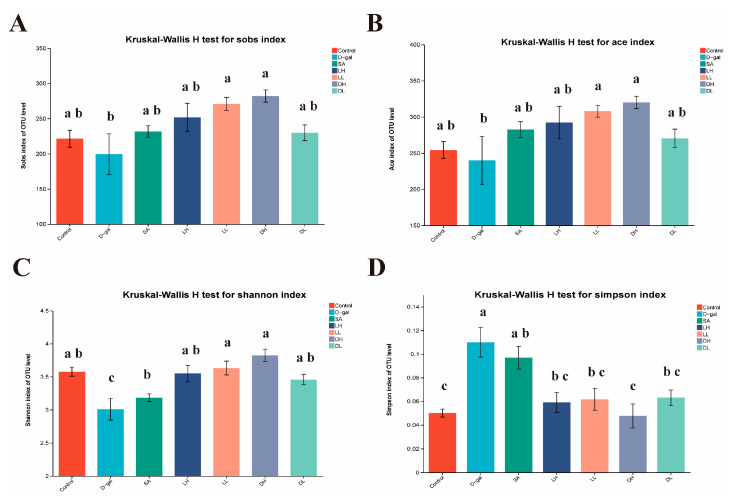
Impact of *L. plantarum* TY-Y10 on alpha diversity of the gut microbiota in aging mice. (**A**) Sobs index of OTU level; (**B**) Ace index of OTU level; (**C**) Shannon index of OTU level; (**D**) Sipmson index of OTU level. The data are expressed as mean ± SD. Statistical significance was assessed using ANOVA, and differences were considered significant at *p* < 0.05. Groups that differed significantly are indicated by different letters. Control, control group; D-gal, model group; SA, positive drug group; LH, high dose of viable and *L. plantarum* TY-Y10; LL, low dose of viable *L. plantarum* TY-Y10; DH, high dose of heat-inactivated *L. plantarum* TY-Y10; DL, low dose of heat-inactivated *L. plantarum* TY-Y10.

To determine if the effects depicted in Figure 6 and Figure 7 were influenced by changes in the intestinal bacterial community, gut microbiome profiling was conducted for all mice. The alpha diversity of the gut microbiota was assessed using four key indices to evaluate the influence of *L. plantarum* TY-Y10. As illustrated in Figure 8, the Sob, Ace, and Shannon indices significantly decreased in the D-gal group compared to the control group, while the Simpson index showed a notable increase. However, in the *L. plantarum* TY-Y10 intervention group, these indices showed significant improvement (*p* < 0.05). To some extent, the observed diversity changes support the potential role of *L. plantarum* TY-Y10 in improving the composition of the intestinal microbiota in mice.

**Figure 9:**
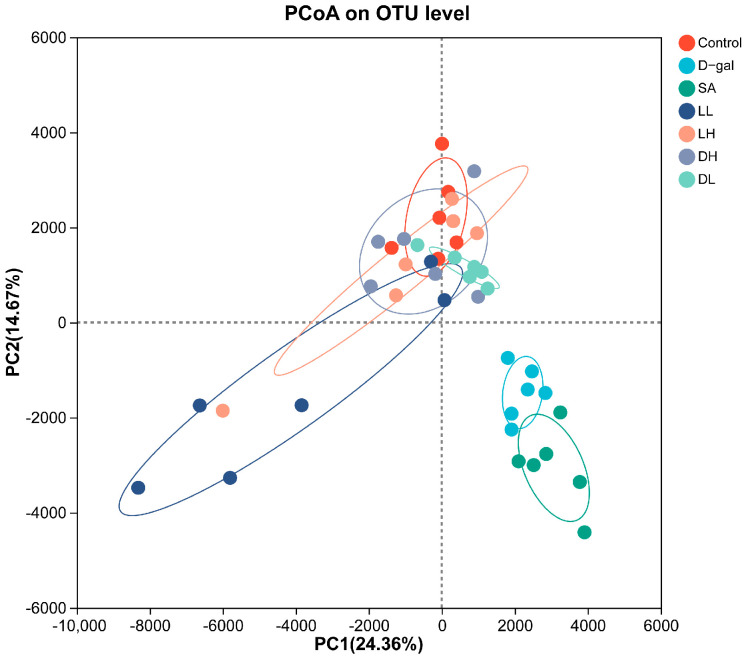
Impact of *L. plantarum* TY-Y10 on beta diversity of the gut microbiota in aging mice. Control, control group; D-gal, model group; SA, positive drug group; LH, high dose of viable and *L. plantarum* TY-Y10; LL, low dose of viable *L. plantarum* TY-Y10; DH, high dose of heat-inactivated *L. plantarum* TY-Y10; DL, low dose of heat-inactivated *L. plantarum* TY-Y10.

To further evaluate the differences in microbial composition and beta diversity of the gut microbiota at the OTU level across all groups, we conducted a principal coordinate analysis (PCoA) based on Euclidean distances. As depicted in Figure 9, there was a distinct separation between the control group and the D-gal group, reflecting significant compositional differences. However, in the groups treated with *L. plantarum* TY-Y10, the microbial profiles showed a shift towards the control group, suggesting that *L. plantarum* TY-Y10 supplementation contributed to a reconfiguration of the gut microbiota composition in aging mice.

**Figure 10:**
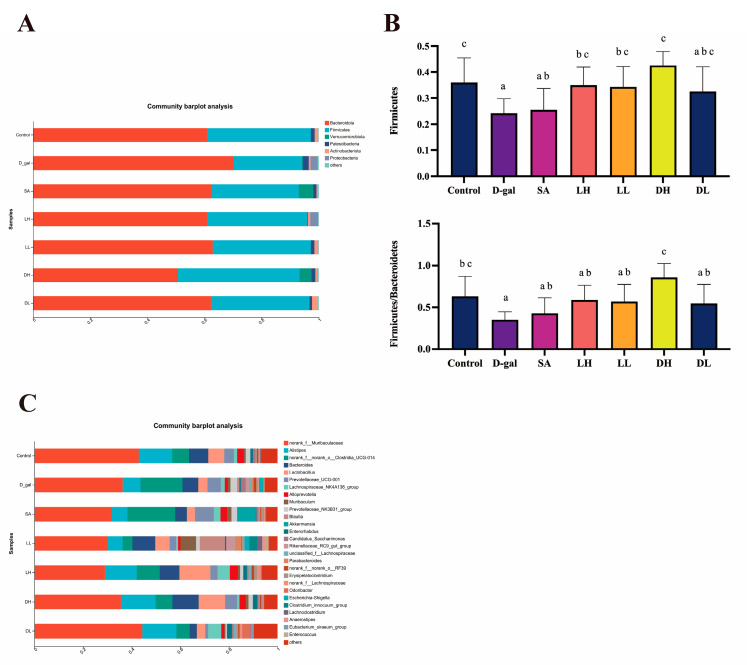
Impact of *L. plantarum* TY-Y10 on gut microbiota composition in aging mice. (**A**) The bar plot for different groups of main phyla abundance; (**B**) phylum-level analysis of Firmicutes abundance and Firmicutes-to-Bacteroidetes ratio in gut microbiota; (**C**) the bar plot for different groups of genera with abundance ≥1%. The data are expressed as mean ± SD. Statistical significance was assessed using ANOVA, and differences were considered significant at *p* < 0.05. Groups that differed significantly are indicated by different letters. Control, control group; D-gal, model group; SA: positive drug group; LH, high dose of viable and *L. plantarum* TY-Y10; LL, low dose of viable *L. plantarum* TY-Y10; DH, high dose of heat-inactivated *L. plantarum* TY-Y10; DL, low dose of heat-inactivated *L. plantarum* TY-Y10.

Based on the previously discussed findings, we performed a detailed analysis of the differential abundance of gut microbiota at both the phylum and genus levels in the various mice groups, as classified according to OTU taxonomy. These results are presented in Figure 10. At the phylum level, the gut microbiota across all groups included Bacteroidetes, Firmicutes, Verrucomicrobia, Actinobacteria, Patescibacteria, and Proteobacteria (Figure 10A). Our analysis particularly focused on Firmicutes and the Firmicutes-to-Bacteroidetes ratio, as these are known to be associated with aging processes. As shown in Figure 10B, D-gal induction led to a decrease in the relative abundance of Firmicutes and the Firmicutes-to-Bacteroidetes ratio, while the abundance of Bacteroidetes increased. Notably, these alterations were reversed following intervention with *L. plantarum* TY-Y10, indicating a restoration towards a more balanced gut microbiota composition.

As shown in Figure 10C, at the genus level, we analyzed bacteria with a relative abundance of more than 1%, including norank_f_Muribaculaceae, *Lactobacillus*, norank_f_norank_o_Clostridia_UCG-01 *Alistipe*, *Bacteroides*, *Prevotellaceae*_UCG-001, *Lachnospiraceae*_NK4A136_group, *Muribaculum*, *Alloprevotella*, *Muribaculum*, *Prevotellaceae*_NK3B31_group, *Blautia*, *Akkermansia*, *Enterorhabdus*, *Candidatus*_*Saccharimonas*, *Rikenellaceae*_RC9_gut_group, unclassified_f__*Lachnospiraceae*, *Parabacteroides*, norank_f__norank_o__RF39, *Erysipelatoclostridium*, norank_f__*Lachnospiraceae*, *Odoribacter*, *Escherichia-Shigella*, *Clostridium*_*innocuum*_group, *Lachnoclostridium*, *Anaerostipes*, *Eubacterium*_*siraeum*_group, and *Enterococcus*.

**Figure 11:**
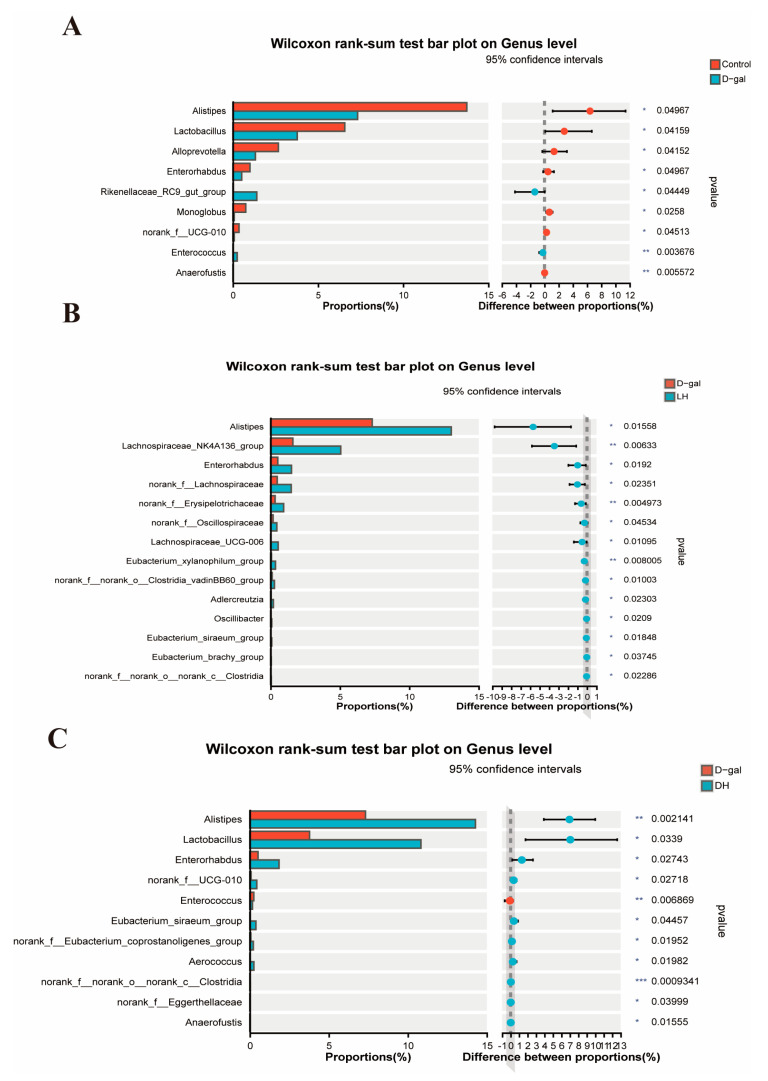
Differential genus-level gut microbiota composition in aging mice following *L. plantarum* TY-Y10 intervention. (**A**) The differences in gut microbiota composition on genus level between the control group and D-gal group; (**B**) the differences in gut microbiota composition on genus level between LH group and D-gal group; (**C**) the differences in gut microbiota composition on genus level between DH group and D-gal group. * indicates significance (* *p* < 0.05; ** *p* < 0.01; *** *p* < 0.001). Control, control group; D-gal, model group; SA, positive drug group; LH, high dose of viable and *L. plantarum* TY-Y10; LL, low dose of viable *L. plantarum* TY-Y10; DH, high dose of heat-inactivated *L. plantarum* TY-Y10; DL, low dose of heat-inactivated *L. plantarum* TY-Y10.

To evaluate variations in intestinal microbial composition at the genus level across different experimental groups, we employed the Wilcoxon rank-sum test. The analysis revealed significant alterations in the gut microbiota profiles induced by D-gal. Furthermore, the intervention with *L. plantarum* TY-Y10 significantly modified these compositional changes. As shown in Figure 11A, following the administration of D-gal, the abundance of *Alistipes*, *Lactobacillus*, *Alloprevotella*, *Enterorhabdus*, *Monoglobus*, norank_f_UCG-010, and *Anaerofustis* was significantly decreased, whereas *Rikenellaceae_RC9_gut_group* and *Enterococcus* significantly increased. Figure 11B shows the intervention of viable *L. plantarum* TY-Y10 increased the abundance of *Alistipes*, *Lachnospiraceae_NK4A136_group*, *Enterorhabdus*, norank_f_*Lachnospiraceae*, norank_f_*Erysipelotrichaceae*, norank_f_*Oscillospiraceae*, *Lachnospiraceae*_UCG_006, *Eubacterium_xylanophilum*_group, *norank_f_norank_o_Clostridia_vadinBB60_group*, *Adlercreutzia*, *Oscillibacter*, *Eubacterium_siraeum_group*, *Eubacterium_brachy*_group, and *norank_f_norank_o_norank_c_Clostridia* was significantly increased. Similarly, heat-inactivated *L. plantarum* TY-Y10 significantly raised the abundance of *Alistipes*, *Lactobacillus*, *Enterorhabdus*, *norank_f_UCG-010*, *Enterococcus*, *Eubacterium_siraeum_group*, *norank_f_Eubacterium_coprostanoligenes_group*, *Aerococcus*, *norank_f_norank_o_norank_c_Clostridia*, *norank_f_Eggerthellaceae*, and *Anaerofustis* (Figure 11C).

### 3.6. L. plantarum TY-Y10 Increases SCFA Level in Aging Mice

**Figure 12:**
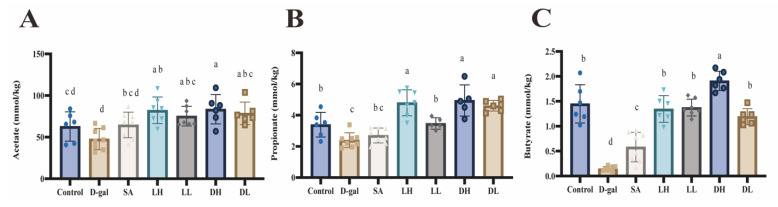
Impact of *L. plantarum* TY-Y10 on SCFA level in aging mice. (**A**) Acetate level; (**B**) propionate level; (**C**) butyrate level. The data are expressed as mean ± SD. Statistical significance was assessed using ANOVA, and differences were considered significant at *p* < 0.05. Groups that differed significantly are indicated by different letters. Control, control group; D-gal, model group; SA, positive drug group; LH, high dose of viable and *L. plantarum* TY-Y10; LL, low dose of viable *L. plantarum* TY-Y10; DH, high dose of heat-inactivated *L. plantarum* TY-Y10; DL, low dose of heat-inactivated *L. plantarum* TY-Y10.

The levels of SCFAs in feces quantified using GC are shown in Figure 12. We conducted a targeted analysis of three SCFAs. The results showed that D-gal administration downregulated fecal acetate, propanoate, and butyrate, while *L. plantarum* TY-Y10 played a significant upregulation role.

## 4. Discussion

The underlying mechanisms and the preventive approach to aging have been extensively studied. Population aging has led to a global increase in chronic diseases, posing a significant challenge to society [30]. Aging is closely linked to disrupted redox balance and is characterized by the accumulation of oxidative damage at the cellular level [31]. The supplementation of exogenous antioxidants to attenuate oxidative stress-induced aging is widely recognized. Our study focused on developing novel, effective, and safe functional food for alleviating oxidative stress. Studies on gut microbial homeostasis as an important hallmark of human aging have identified gut microbiota as a new target for modulation to encourage increased systemic antioxidant levels to delay aging [32], with probiotics emerging as a particularly promising source. We discovered a potential antioxidant strain, *L. plantarum* TY-Y10, from our previous studies on the screening of 47 Lactobacillus strains based on reducing power, DPPH radical scavenging, and hydroxyl radical scavenging activity assays in vitro. Additionally, the *L. plantarum* TY-Y10 exhibited a survival rate of 102.68% after 3 h of culture at pH = 3, and a survival rate of 100.23% after 8 h of culture in a bile salt concentration of 0.3%. Compared to other lactic acid bacteria, it demonstrated the excellent capacity to withstand acid and bile salts stress [33]. In this study, we conducted an in vivo experiment to comprehensively investigate the antioxidant effects of *L. plantarum* TY-Y10. The result showed that *L. plantarum* TY-Y10 affected the intestinal flora and intestinal metabolites of mice and further activated the Nrf2 signaling pathway to alleviating the liver oxidative stress in aging mice.

The D-gal-induced aging model is widely utilized in oxidative stress research due to its effectiveness in simulating aging-related oxidative damage. D-gal induces oxidative stress primarily through two distinct pathways. In the first pathway, D-gal increases the production of reactive oxygen species (ROS) by enhancing NADPH oxidase activity, which subsequently generates aldehydes and hydrogen peroxide via a galactose oxidase-catalyzed reaction. Another pathway through which D-galactose induces oxidative stress involves an increase in galactitol caused by excessive intake of D-gal under the action of galactose reductase. This galactitol cannot be metabolized normally, resulting in abnormal quantities that cause osmotic stress, thereby impairing the antioxidant system [34]. Numerous studies have confirmed that D-gal administration causes injury across multiple organs, with the liver being particularly vulnerable due to its role as the primary metabolic organ with a high metabolic rate, making it more susceptible to ROS damage [35,36,37]. In our study, a significant reduction in liver index was observed in the D-gal group compared to the normal control (NC) group, likely reflecting liver pathology. This finding suggests that prolonged exposure to D-gal contributes to liver damage. The underlying mechanism is believed to involve excessive ROS generation from high-dose D-gal, which damages various biomolecules, including lipids, proteins, and DNA, ultimately leading to cellular damage and accelerated aging [38].

MDA serves as a direct indicator of lipid peroxidation and is widely recognized as a reliable biomarker for assessing oxidative stress in clinical settings [39]. GSH, belonging to the non-antioxidant enzyme system, is synthesized from three components: cysteine, glutamate, and glycine. The cysteine sulfhydryl group in GSH is critical for its antioxidant properties and its ability to participate in reduction and conjugation reactions [40]. SOD is an essential antioxidant enzyme that scavenges superoxide anions by catalyzing their dismutation into hydrogen peroxide, which is subsequently broken down into water [41]. CAT is an essential enzyme in the body’s defense against oxidative stress. It catalyzes the conversion of hydrogen peroxide, a harmful byproduct of cellular metabolism, into water and oxygen [42]. GSH-PX, with the selenocysteine active center, catalyzes the reduction of excess hydrogen peroxide to harmless hydroxyl compounds using GSH [43]. In this study, all the above indicators in both the liver and serum were tested. The findings revealed that treatment with either viable or heat-inactivated *L. plantarum* TY-Y10 significantly enhanced the antioxidant defense mechanisms, as evidenced by the increased activities of SOD, CAT, GSH-PX, and elevated levels of GSH. Additionally, there was a notable reduction in MDA levels, suggesting that *L. plantarum* TY-Y10 effectively mitigates oxidative stress in aging mice.

Research has demonstrated that prolonged oxidative stress can activate chronic inflammatory pathways, leading to the production and release of pro-inflammatory cytokines [44]. Simultaneously, inflammation contributes to increased ROS production, which exacerbates oxidative stress and intensifies the inflammatory response [45]. This reciprocal relationship between oxidative stress and inflammation can create a damaging feedback loop that worsens liver injury. To investigate this, we evaluated the impact of *L. plantarum* TY-Y10 on serum levels of IL-6, TNF-α, and IFN-γ. The findings indicated that treatment with both viable and heat-inactivated *L. plantarum* TY-Y10 not only mitigated oxidative damage in aging mice but also significantly reduced the elevated pro-inflammatory cytokine levels induced by D-gal. This prompted us to further explore the protective effects of *L. plantarum* TY-Y10 on liver health through histological analysis. Consistent with our expectations, *L. plantarum* TY-Y10 alleviated the hepatic pathological changes caused by D-gal.

Nrf2 is a crucial transcription factor that mitigates oxidative stress by directly enhancing the expression of various antioxidant and detoxification genes [46]. The role of Nrf2 in combating oxidative stress has been well-documented, emphasizing its importance in cellular defense mechanisms [47,48,49]. In this study, activating the Nrf2 signaling pathway might be a potential mechanism through which *L. plantarum* TY-Y10 alleviated oxidative stress and protected the liver during mice aging. This mechanism was confirmed by our assay of the Nrf2 signaling pathway at both the protein and mRNA levels in the mice’s liver. Consistent with previous research, administration of D-gal led to a significant downregulation of Nrf2 expression in the liver of aging mice, which subsequently resulted in decreased levels of downstream antioxidant proteins. Conversely, treatment with *L. plantarum* TY-Y10, especially in its heat-inactivated form, notably elevated the expression of Nrf2 and its associated target proteins. These observations suggest that *L. plantarum* TY-Y10 exerts its protective effects against oxidative stress and liver damage through the activation of the Nrf2 signaling pathway, thereby enhancing the antioxidant defense mechanisms in aging mice subjected to D-gal.

To investigate the mechanisms through which *L. plantarum* TY-Y10 supplementation influences oxidative damage in the liver by activating the Nrf2 signaling pathway, we postulated that alterations in gut microbiota composition and host metabolism, mediated via the gut-liver axis, might be involved. We employed 16S rDNA sequencing to analyze the changes in gut microbiota diversity and composition following *L. plantarum* TY-Y10 supplementation. Our analysis revealed notable alterations in both alpha and beta diversity metrics. Specifically, D-gal led to a significant reduction in the diversity and richness of the gut microbiota. Conversely, supplementation with *L. plantarum* TY-Y10 effectively restored these parameters, suggesting a beneficial modulation of gut microbiota structure. At the phylum level, D-gal resulted in a notable decline in the relative abundance of Firmicutes and a reduction in the Firmicutes/Bacteroidetes ratio, whereas *L. plantarum* TY-Y10 returned them to normal levels. These findings are consistent with previous studies by Zhang et al., who observed a similar decline in the gut microbiota of aged mice [50]. The reduction in Firmicutes has also been documented in patients with age-related conditions such as Alzheimer’s and Parkinson’s diseases [51,52]. Furthermore, studies have demonstrated a positive correlation between the Firmicutes/Bacteroidetes ratio and short-chain fatty acid (SCFA) concentrations [53,54]. At the genus level, a comparative analysis of gut microbiota between the control and D-gal groups revealed substantial alterations. Specifically, D-gal treatment led to a significant reduction in the abundance of beneficial bacteria while promoting the proliferation of potentially harmful bacteria within the intestines of aging mice. This intervention also resulted in a marked decrease in the populations of bacteria responsible for producing short-chain fatty acids SCFAs. Notably, the genus *Alistipes*, a key SCFA producer, was found to be significantly diminished in the D-gal group [55]. The genus *Lactobacillus*, known for its beneficial roles including the synthesis of neurotransmitters and metabolites such as serotonin, histamine and SCFAs, was also affected. These bacteria contribute to maintaining gut homeostasis by acidifying the intestinal environment and suppressing the growth of pathogenic microorganisms [56]. *Alloprevotella* is a short-chain fatty acid-producing bacterium. Zhao et al. reported that metformin alleviated neuroinflammation associated with sepsis by regulating the abundance of *Alloprevotella* in the intestine and then regulating the intestinal metabolite SCFAs [57]. Ge et al. [58] found that the increases in the abundances of *Alistipes*, *Lactobacillus*, and *Alloprevotella* increased SCFA contents in feces. In addition, *Monoglobus* abundance has been shown to be positively correlated with dietary fiber fermentation and bile acid metabolism, which is associated with healthy communities [59]. *Anaerofustis* is a beneficial bacterium. Wang et al. pointed out that *Anaerofustis*, as a potential short-chain fatty acid producer, might be related to the remission of COVID-19 pneumonia [60]. Among the bacteria with significantly increased abundance in the D-gal group, *Rikenellace_RC9_gut_group* was positively associated with psychiatric disorders [61]. An increase in its abundance has also been reported in another study of D-gal-induced oxidative stress mice [62]. *Enterocloster* was more abundant in mice with lower histopathological scores, which was associated with infections such as bacteremia [63,64]. *L. plantarum* TY-Y10 demonstrated a dose-dependent effect in mitigating liver oxidative damage in aging mice. Detailed analysis of gut microbiota alterations between the D-gal group and the high-dose groups of both viable and heat-inactivated *L. plantarum* TY-Y10 revealed notable differences. Specifically, both viable and heat-inactivated *L. plantarum* TY-Y10 interventions led to a significant increase in the abundance of bacteria that produce SCFAs. This includes the genera *Alistipes* and *Lactobacillus*, as well as the *Lachnospiracea* family, particularly the *Lachnospiracea_NK4A136_group*, known for its SCFA production. The increased presence of *Lachnospiracea_NK4A136_group* has been linked to improvements in intestinal barrier integrity and microbiota function, as evidenced by its significant correlation with enhanced intestinal barrier function in studies involving arginine supplementation in diet-induced obese mice [65]. In investigating the impact of *L. plantarum* TY-Y10 on the gut microbiota of aging mice, our study found that both viable and heat-inactivated forms of *L. plantarum* TY-Y10 effectively mitigated oxidative damage associated with aging. This protective effect was primarily achieved by counteracting the reduction in populations of SCFA-producing bacteria. This finding was further corroborated by subsequent analyses of SCFA levels in mouse feces, conducted using gas chromatography (GC).

SCFAs, produced by the gut microbiota, are known for their anti-inflammatory effects and anti-proliferative effects on cancer or tumor cells, which are closely linked to redox signaling mechanisms [66,67]. Evidence from several studies suggests that SCFAs provide protection against oxidative and mitochondrial stress, indicating their potential as both nutritional and therapeutic agents for promoting healthy aging [68]. In our targeted analysis of fecal acetate, propanoate, and butyrate, it was observed that *L. plantarum* TY-Y10 significantly elevated the levels of SCFAs reduced by D-gal, particularly butyrate. Butyric acid, the predominant SCFA, serves as a critical energy source for colonocytes. Studies have shown that SCFA plays an antioxidant role mainly through butyric acid, which serves as a regulator of Nrf2-Keap1 signaling and promotes Nrf2 nuclear translocation [69,70,71]. Furthermore, butyrate can migrate from the intestinal tract to the liver via the portal vein, activating Nrf2, which plays an important role in the host gut–liver axis [72].

## 5. Conclusions

In this study, *L. plantarum* TY-Y10 regulated SCFAs and the metabolite of the gut microbiota by regulating the composition of gut microbiota. SCFAs activated the liver Nrf2-Keapl signaling pathway through the gut–liver axis. In addition, they upregulated the expression of antioxidant genes and alleviated oxidative damage in the mouse liver. However, the role and mechanism of butyric acid in activating the Nrf2 signaling pathway to alleviate liver oxidative damage need further analysis. This study reveals the effect of *L. plantarum* TY-Y10 in alleviating oxidative damage during the aging process in vivo. It also provides evidence to verify the characteristics and effects of probiotics in delaying aging and clarifies the mechanism of action to a certain extent.

## Figures and Tables

**Table 1:** Primer list.

Gene Name	Accession Number	Primer Sequence/(5′-3′)
F-Nrf2	NM_010902.5	CAGTGCTCCTATGCGTGAA
R-Nrf2		GCGGCTTGAATGTTTGTC
F-Keap1	XM_029481764.1	CACACTAGAGGATCACACCAAG
R-Keap1		CCGTGTAGGCGAACTCAATAA
F-CAT	NM_009804	CGTTCGATTCTCCACAGTCA
R-CAT		CCCACAAGATCCCAGTTACC
F-SOD2	NM_013671.3	TAACGCGCAGATCATGCAGCTG
R-SOD2		AGGCTGAAGAGCGACCTGAGTT
F-GPX-1	NM_001329528.1	AAGGCTCACCCGCTCTTTAC
R-GPX-1		ACACCGGAGACCAAATGATG

**Table 2:** Impact of *L. plantarum* TY-Y10 supplementation on organ indices in aging mice. Statistical significance was assessed using ANOVA, and differences were considered significant at *p* < 0.05. Groups that differed significantly are indicated by different letters.

Group	Liver Index (mg/g)	Kidney Index (mg/g)	Spleen Index (mg/g)
Control	43.73 ± 1.34 ^a^	14.52 ± 0.47	3.20 ± 0.15
D-gal	34.79 ± 0.91 ^b^	13.82 ± 0.43	2.93 ± 0.12
SA	40.14 ± 2.36 ^a^	14.14 ± 0.35	3.12 ± 0.13
LH	43.10 ± 3.12 ^a^	14.14 ± 0.66	3.19 ± 0.11
LL	40.68 ± 1.51 ^a^	14.04 ± 0.42	3.18 ± 0.10
DH	43.99 ± 2.61 ^a^	14.37 ± 0.48	3.21 ± 0.16
DL	42.39 ± 2.38 ^a^	14.16 ± 0.57	3.19 ± 0.09

## Data Availability

The original contributions presented in the study are included in the article, further inquiries can be directed to the corresponding authors.
